# Editorial: Transcription factors in immunological disease and haematological malignancies

**DOI:** 10.3389/fimmu.2024.1413841

**Published:** 2024-05-31

**Authors:** Jacob T. Jackson, Ashley P. Ng, Stephen L. Nutt, Tomokatsu Ikawa, Li Wu

**Affiliations:** ^1^ Immunology Division, Walter and Eliza Hall Institute of Medical Research, The University of Melbourne, Melbourne, VIC, Australia; ^2^ Division of Immunology and Allergy, Research Institute for Biomedical Sciences, Tokyo University of Science, Tokyo, Japan; ^3^ School of Medicine, Tsinghua University, Beijing, China

**Keywords:** transcription factors, HHEX, KLF2, ZHX2, epithelial to mesenchymal transformation (EMT), NF- kappa B, CDKN2A, RBP-J

Our Research Topic, delves into key transcription factors involved in immunological diseases and haematological malignancies, and brings to the fore cutting-edge research and thorough and targeted literature reviews. These timely works highlight the future potential of targeting transcription factors for clinical intervention in the treatment of a range of diseases in which they may be critically involved.

With regards to the original research articles, Butcher et al. employed elegant mouse models to demonstrate the T-bet+ Th17 cells, responsible for experimental autoimmune encephalitis through induction of GM-CSF, are governed by expression of GATA3 which drives expression of Egr2, Bhlhe40, and Csf2. Liang et al. reveal that RBP-J–mediated Notch signalling regulates macrophage development and activation. Their murine Parkinson’s Disease (PD) model showed Notch signalling within microglia resulted in decreased tyrosine hydroxylase positive neurons that was blocked by inactivation of RBP-J that decreased infiltrating, inflammatory macrophages and activated microglia. This work showed for the first time that RBP-J–mediated Notch signalling may well play a significant role the development of PD predominantly through the regulation of the activation of microglia via NF-κB signalling. Research presented by Mah et al. reports on the important role of ING5 for normal liver cellularity in foetal development in a cell extrinsic fashion using a gene knockout mouse model. However, a third of these mice survived weaning and ING5 was not found to be required for haematopoietic stem cell self-renewal. Interestingly, the highly related ING4 transcription factor, bearing an identical homeodomain may provide some level of redundancy. Trezise et al. explored the results of a primary B cell CRISPR/Cas9-mediated screen, which illuminated key components of the pathway mediating antibody secretion. The results of these studies identifies potential candidates may be targeted for clinical treatment for antibody-mediated diseases, and potential pathogenic genes that may underlie primary antibody deficiencies. These highlight the importance of discovery research into the functional mechanisms of transcription factors in normal and disease development, which can inform future diagnostic and therapeutic strategies.

Our Research Topic also brings together a number of in-depth scientific reviews of the literature. The first is provided by Balendran et al. with a focus on the transcription factors, NF-κB, STATs, AP-1 and IRFs with regards to their critical role in inflammatory disease. These transcription factors may serve as potential therapeutic targets in rheumatoid arthritis, through targeting with direct inhibitors, or via targeting signalling pathways that may activate these transcription factors, or exploring transcription factor interaction with a natural compound screen.

Jackson et al. delivers a comprehensive review of the role of Hhex in development, physiology and disease, where the pleiotrophic actions of Hhex have been shown to be dependent on the cellular context. Salient observations include how the function of Hhex in embryological development can be reflected in disease processes that may involve Hhex, including repression of *Cdkn2a* in the context of HSC self-renewal, emergency haematopoiesis and acute myeloid leukaemia, as well as potential roles in type 2 diabetes where both *HHEX* and *CDKN2A* variants very frequently occur together as genetic risk factors.

The literature behind the current state of knowledge for ZHX2 in normal cellular processes, including proliferation, differentiation, and metabolism homeostasis, was explored by Li et al. The involvement of ZHX2 in cancer is also reviewed with its potential role as an oncogene in hepatocellular carcinoma, clear cell renal cell carcinoma and triple-negative breast cancer increasingly recognised.

Radhakrishan et al. present an in-depth review on the role of epithelial-mesenchymal transition (EMT) transcription factors of the ZEB, TWIST and SNAIL families. These transcription factors are important in haematopoiesis, with roles in haematological malignancy increasingly recognised. Such oncogenic roles have become evident with overexpression linked to worse clinical outcomes in myeloid malignancies, with dysregulation, mutation and chromosomal aberrations involving these factors also observed in lymphoid neoplasms. Unlike Hhex, EMT transcription factor roles in haematopoiesis have been suggested to be broadly distinct from roles in embryological development.

The review by Roy et al. examines proliferation of B cells and their germinal centre development, including generation of plasmablasts and plasma cells is governed by the signalling of NF-κB. They also detail how NF-κB monomers each serve in specific roles in the differentiation/formation of plasma cells and germinal centre B cells. This body of research helps inform a number of B cell-driven diseases, including lymphoma, immunodeficiency and autoimmunity, as well as importantly allowing the authors to pose a number of outstanding questions in field.

The role of the transcription factor KLF2 in B cells, specifically in terms of development, activation, generation and maintenance of plasma cells, is reviewed by Wittner and Schuh. This report explores the function of KLF2 as both an activator and an inhibitor of various B cell functions, depending on immunological context, as well as describing KLF2’s known roles in B cell malignancies such as multiple myeloma and splenic marginal zone lymphoma, and diseases such as IgA deficiencies.

Finally, Zhang et al.‘s review explores the growing literature around dendritic cell (DC) differentiation. The heterogenicity of type 2 conventional DCs (cDC2), the origins of plasmacytoid DCs (pDCs), and emerging knowledge on DC3, the latter of which share features of both cDC2s and monocyte-derived DCs, is specifically explored. Insights into the transcription factors governing these cell types including IRF8, PU.1 and E2–2 and provided. The data presented in their review suggest that the development of cDCs and pDCs relies heavily on a balance between several key transcription factor pairs, notably E2.2/ZEB2 versus ID2/NFIL3 or PU.1 versus BCL11A.

Collectively, these publications shine a light on the crucial roles of a number of key transcription factors across normal development, as well as immunological and haematological malignancy. The publications highlighted in this section strongly validates the need for discovery research that can yield important new insights and novel therapeutic approaches in the treatment of diseases of unmet clinical need.

## Author contributions

JJ: Writing – review & editing, Writing – original draft, Supervision, Project administration, Conceptualization. AN: Writing – review & editing. SN: Writing – review & editing, Supervision, Funding acquisition. TI: Writing – review & editing. LW: Writing – review & editing.

